# 2-Methyl-6-(6-methyl-1*H*-benzimidazol-2-yl)phenol–2-methyl-6-(5-methyl-1*H*-benzimidazol-2-yl)phenol (3/1)

**DOI:** 10.1107/S1600536809049770

**Published:** 2009-11-28

**Authors:** Naser Eltaher Eltayeb, Siang Guan Teoh, Rohana Adnan, Hoong-Kun Fun, Suchada Chantrapromma

**Affiliations:** a School of Chemical Science, Universiti Sains Malaysia, 11800 USM, Penang, Malaysia; b X-ray Crystallography Unit, School of Physics, Universiti Sains Malaysia, 11800 USM, Penang, Malaysia; c Crystal Materials Research Unit, Department of Chemistry, Faculty of Science, Prince of Songkla University, Hat-Yai, Songkhla 90112, Thailand

## Abstract

The title compound, 0.75C_15_H_14_N_2_O·0.25C_15_H_14_N_2_O, is a co-crystal of 2-methyl-6-(6-methyl-1*H*-benzimidazol-2-yl)phenol as the major component and 2-methyl-6-(5-methyl-1*H*-benz­imidazol-2-yl)phenol as the minor component. The refined site-occupancy ratio is 0.746 (4)/0.254 (4). The conformations of both components are identical except for that of the methyl substituent on the benzene ring of the benzimidazole unit which is positionally disordered over two positions. The mol­ecule is essentially planar, the dihedral angle between the benzimidazole plane and the benzene ring being 3.49 (4)°. An intra­molecular O—H⋯N hydrogen bond generates an *S*(6) ring motif. In the crystal packing, mol­ecules are linked through N—H⋯O hydrogen bonds into chains along [201]. These chains are stacked approximately along the *a*-axis direction. The crystal packing is further stabilized by weak N—H⋯O and O⋯H⋯N hydrogen bonds, together with weak inter­molecular C—H⋯π inter­actions. A π–π inter­action with a centroid–centroid distance of 3.6241 (6) Å is also observed between the substituted phenyl ring and that of the benzimidazole system.

## Related literature

For bond-length data, see: Allen *et al.* (1987 ▶). For hydrogen-bond motifs, see: Bernstein *et al.* (1995 ▶). For background to benzimidazoles and their bioactivity, see: Demirayak *et al.* (2002 ▶); Guven *et al.* (2007 ▶); Minoura *et al.* (2004 ▶); Pawar *et al.* (2005 ▶); Thakurdesai *et al.* (2007 ▶); Tomei *et al.* (2003 ▶). For related structures, see: Eltayeb *et al.* (2007 ▶, 2009*a* ▶,*b* ▶); Xiao *et al.* (2009 ▶). For the stability of the temperature controller used in the data collection, see Cosier & Glazer, (1986 ▶).
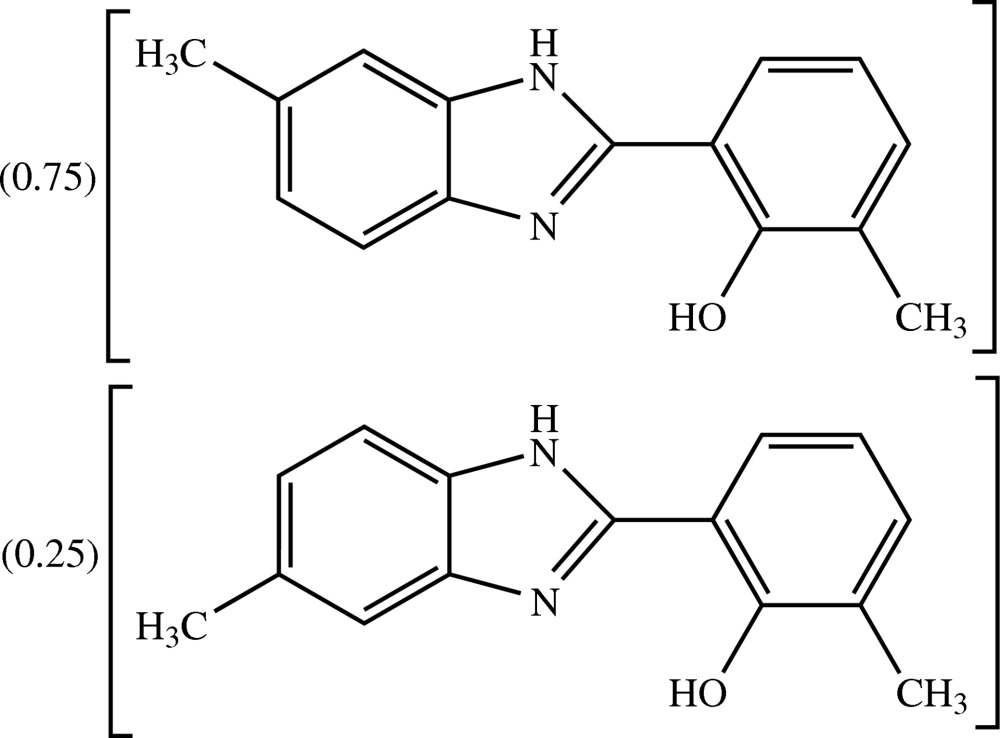


## Experimental

### 

#### Crystal data

0.75C_15_H_14_N_2_O·0.25C_15_H_14_N_2_O
*M* *_r_* = 238.28
Monoclinic, 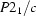

*a* = 4.9231 (1) Å
*b* = 19.8900 (6) Å
*c* = 12.3199 (3) Å
β = 105.085 (1)°
*V* = 1164.80 (5) Å^3^
*Z* = 4
Mo *K*α radiation
μ = 0.09 mm^−1^
*T* = 100 K
0.59 × 0.17 × 0.10 mm

#### Data collection

Bruker APEXII CCD area-detector diffractometer
Absorption correction: multi-scan (*SADABS*; Bruker, 2005 ▶) *T*_min_ = 0.951, *T*_max_ = 0.992
34431 measured reflections
3703 independent reflections
3165 reflections with *I* > 2σ(*I*)
*R*_int_ = 0.030

#### Refinement

*R*[*F* ^2^ > 2σ(*F* ^2^)] = 0.046
*wR*(*F* ^2^) = 0.131
*S* = 1.07
3703 reflections
184 parameters
H atoms treated by a mixture of independent and constrained refinement
Δρ_max_ = 0.61 e Å^−3^
Δρ_min_ = −0.20 e Å^−3^

### 

Data collection: *APEX2* (Bruker, 2005 ▶); cell refinement: *SAINT* (Bruker, 2005 ▶); data reduction: *SAINT*; program(s) used to solve structure: *SHELXTL* (Sheldrick, 2008 ▶); program(s) used to refine structure: *SHELXTL*; molecular graphics: *SHELXTL*; software used to prepare material for publication: *SHELXTL* and *PLATON* (Spek, 2009 ▶).

## Supplementary Material

Crystal structure: contains datablocks global, I. DOI: 10.1107/S1600536809049770/sj2688sup1.cif

Structure factors: contains datablocks I. DOI: 10.1107/S1600536809049770/sj2688Isup2.hkl

Additional supplementary materials:  crystallographic information; 3D view; checkCIF report

## Figures and Tables

**Table 1:** Hydrogen-bond geometry (Å, °)

*D*—H⋯*A*	*D*—H	H⋯*A*	*D*⋯*A*	*D*—H⋯*A*
N2—H1*N*2⋯O1^i^	0.936 (19)	2.095 (19)	2.9916 (12)	160.1 (18)
O1—H1*O*1⋯N1	0.93 (2)	1.74 (2)	2.6040 (12)	153 (2)
C15—H15*C*⋯*Cg*3^ii^	0.96	2.66	3.5731 (16)	160
C15*A*—H15*F*⋯*Cg*2^iii^	0.96	2.96	3.780 (4)	144

Symmetry codes: (i) 
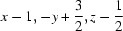
; (ii) 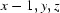; (iii) 
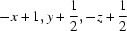
. *Cg*2 and *Cg*3 are the centroids of the C1–C6 and C8–C13 rings, respectively.

**Fig. 1.**
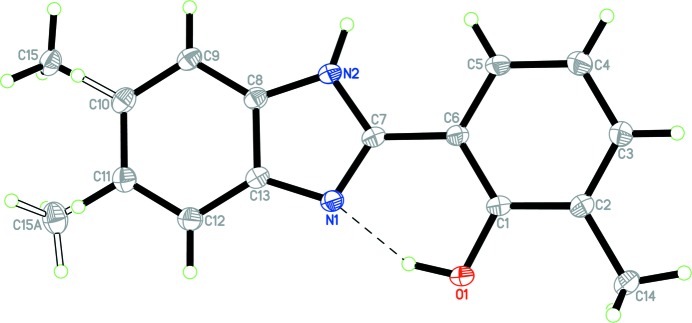
The molecular structure of the title compound, with 50% probability displacement ellipsoids and the atom-numbering scheme. Open bonds show the minor disorder component

**Fig. 2.**
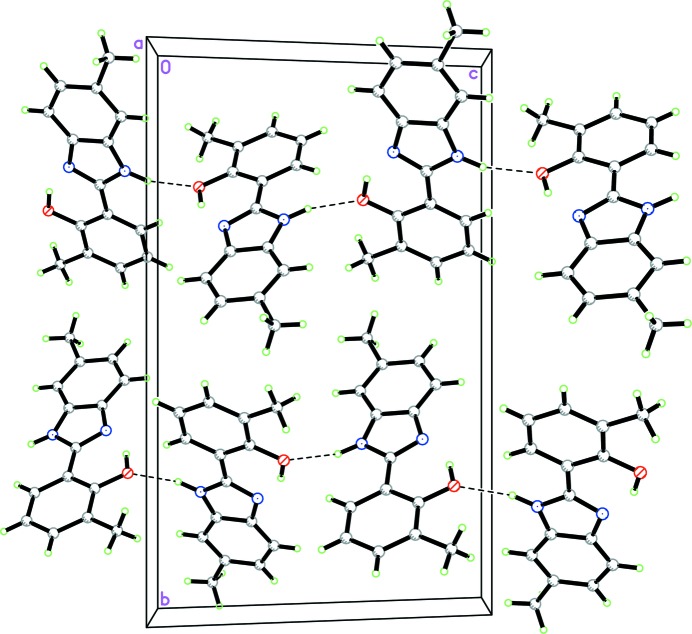
The crystal packing of the major component of the title compound viewed down the a axis. Hydrogen bonds are shown as dashed lines and only the major disorder component is shown.

## supplementary crystallographic information

### Comment

Benzimidazoles are regarded as a promising class of bioactive heterocyclic compounds that exhibit a variety of bioactivities displaying antidiabetic (Minoura *et al.*, 2004), antimicrobial, antifungal (Guven *et al.*, 2007; Pawar *et al.*, 2005), antiviral (Tomei *et al.*, 2003); anti-inflammatory (Thakurdesai *et al*., 2007) and anticancer (Demirayak *et al.*, 2002) properties. As part of our research on screening benzimidazoles for their biological activities, we have previously reported the synthesis and crystal structures of some benzimidazoles (Eltayeb *et al.*, 2007; 2009*a*,*b*). In continuation of this research the title compound (I) was synthesized and its crystal structure is reported here.

The asymmetric unit of the title compound (Fig. 1) consists of [2-methyl-6-(6-methyl-1*H*-benzimidazol-2-yl)phenol] as a major component and [2-methyl-6-(5-methyl-1*H*-benzimidazol-2-yl)phenol] as a minor component. The refined site-occupancy ratio of the major and the minor components is 0.746 (4)/0.254 (4). The conformation of both components are identical except the benzimidazole methyl group is positionally disordered over two positions on atoms C10 and C11 (Fig. 1). The molecule is essentially planar with the dihedral angle between the benzimidazole and benzene ring being 3.49 (4)°. The benzimidazole ring system (N1/N2/C7–C13) is planar with an rms deviation of 0.008 (1)Å. The imidazole ring (N1/N2/C7–C8/C13) makes the dihedral angles of 1.28 (6) and 3.01 (6)° with the C8–C13 and C1–C6 benzene rings, respectively. An intramolecular O—H···N hydrogen bond (Fig. 1) generates an S(6) ring motif (Bernstein *et al.,* 1995) and helps to maintain the planarity of the molecule. Bond lengths in (I) are in normal ranges (Allen *et al.*, 1987) and comparable with those in related structures (Eltayeb *et al.*, 2007; 2009*a*, *b*; Xiao *et al.*, 2009).

The crystal packing of the major component was shown in Fig. 2, the molecules being linked in an antiparallel manner through an N—H···O hydrogen bond into chains along the [2 0 1] direction. These chains are stacked approximately along the *a* axis (Table 1). C—H···π (Table 1) and π–π interactions were also present with the distance Cg_1_···Cg_2_ = 3.6241 (6) Å (symmetry code -1+x, y, z); Cg_1_, Cg_2_ and Cg_3_ are the centroids of the N1/N2/C7–C8/C13, C1–C6 and C8–C13 rings, respectively.

### Experimental

3-Methylsalicylaldehyde (0.5 g, 4 mmol) was added to a solution of 4-methyl-1,2-phenylenediamine (0.244 g, 2 mmol) in ethanol (30 ml). The mixture was refluxed with stirring for half an hour. The resultant yellow solution was filtered. Yellow needle-shaped single crystals of the title compound suitable for *x*-ray structure determination were obtained by slow evaporation of the filtrate at room temperature over several days.

### Refinement

Hydroxy and amide H atoms were located in a difference map and refined isotropically. The remaining H atoms were positioned geometrically and allowed to ride on their parent atoms, with d(C-H) = 0.93 Å for aromatic and 0.96 Å for CH_3_ atoms. The *U*_iso_ values were constrained to be 1.5*U*_eq_ of the carrier atom for methyl H atoms and 1.2*U*_eq_ for the remaining H atoms. A rotating group model was used for the methyl groups. The highest residual electron density peak is located at 0.70 Å from C8 and the deepest hole is located at 1.17 Å from C7. One methyl group is positionally disordered over two positions with occupancies 0.746 (4) (for the methyl group bound to C10) and 0.254 (4) (for the methyl group bound to C11), respectively.

### Crystal data

**Table:**

0.75C_15_H_14_N_2_O·0.25C_15_H_14_N_2_O	*F*(000) = 504
*M**_r_* = 238.28	*D*_x_ = 1.359 Mg m^−^^3^
Monoclinic, *P*2_1_/*c*	Melting point: ?' K
Hall symbol: -P 2ybc	Mo *K*α radiation, λ = 0.71073 Å
*a* = 4.9231 (1) Å	Cell parameters from 3703 reflections
*b* = 19.8900 (6) Å	θ = 2.0–31.0°
*c* = 12.3199 (3) Å	µ = 0.09 mm^−^^1^
β = 105.085 (1)°	*T* = 100 K
*V* = 1164.80 (5) Å^3^	Needle, yellow
*Z* = 4	0.59 × 0.17 × 0.10 mm

### Data collection

**Table:**

Bruker APEXII CCD area-detector diffractometer	3703 independent reflections
Radiation source: sealed tube	3165 reflections with *I* > 2σ(*I*)
graphite	*R*_int_ = 0.030
φ and ω scans	θ_max_ = 31.0°, θ_min_ = 2.0°
Absorption correction: multi-scan (*SADABS*; Bruker, 2005)	*h* = −7→7
*T*_min_ = 0.951, *T*_max_ = 0.992	*k* = −28→28
34431 measured reflections	*l* = −17→17

### Refinement

**Table:**

Refinement on *F*^2^	Primary atom site location: structure-invariant direct methods
Least-squares matrix: full	Secondary atom site location: difference Fourier map
*R*[*F*^2^ > 2σ(*F*^2^)] = 0.046	Hydrogen site location: inferred from neighbouring sites
*wR*(*F*^2^) = 0.131	H atoms treated by a mixture of independent and constrained refinement
*S* = 1.07	*w* = 1/[σ^2^(*F*_o_^2^) + (0.0668*P*)^2^ + 0.4392*P*] where *P* = (*F*_o_^2^ + 2*F*_c_^2^)/3
3703 reflections	(Δ/σ)_max_ = 0.001
184 parameters	Δρ_max_ = 0.61 e Å^−^^3^
0 restraints	Δρ_min_ = −0.20 e Å^−^^3^

### Special details

**Table:**

Experimental. The crystal was placed in the cold stream of an Oxford Cryosystems Cobra open-flow nitrogen cryostat (Cosier & Glazer, 1986) operating at 100.0 (1) K.
Geometry. All esds (except the esd in the dihedral angle between two l.s. planes) are estimated using the full covariance matrix. The cell esds are taken into account individually in the estimation of esds in distances, angles and torsion angles; correlations between esds in cell parameters are only used when they are defined by crystal symmetry. An approximate (isotropic) treatment of cell esds is used for estimating esds involving l.s. planes.
Refinement. Refinement of F^2^ against ALL reflections. The weighted R-factor wR and goodness of fit S are based on F^2^, conventional R-factors R are based on F, with F set to zero for negative F^2^. The threshold expression of F^2^ > 2sigma(F^2^) is used only for calculating R-factors(gt) etc. and is not relevant to the choice of reflections for refinement. R-factors based on F^2^ are statistically about twice as large as those based on F, and R- factors based on ALL data will be even larger.

### Fractional atomic coordinates and isotropic or equivalent isotropic displacement parameters (Å^2^)

**Table:**

	*x*	*y*	*z*	*U*_iso_*/*U*_eq_	Occ. (<1)
O1	1.19279 (16)	0.73306 (4)	0.38129 (6)	0.01798 (17)	
N1	0.75983 (18)	0.80945 (5)	0.29676 (7)	0.01616 (18)	
N2	0.51410 (19)	0.79954 (5)	0.11676 (8)	0.01659 (18)	
C1	1.1529 (2)	0.70454 (5)	0.27765 (8)	0.01503 (19)	
C2	1.3414 (2)	0.65397 (5)	0.26490 (9)	0.0167 (2)	
C3	1.3081 (2)	0.62518 (6)	0.15941 (10)	0.0202 (2)	
H3A	1.4326	0.5918	0.1501	0.024*	
C4	1.0927 (2)	0.64511 (6)	0.06716 (10)	0.0226 (2)	
H4A	1.0756	0.6256	−0.0029	0.027*	
C5	0.9042 (2)	0.69426 (6)	0.08074 (9)	0.0189 (2)	
H5A	0.7591	0.7073	0.0196	0.023*	
C6	0.9303 (2)	0.72445 (5)	0.18570 (8)	0.01520 (19)	
C7	0.7363 (2)	0.77685 (5)	0.20056 (8)	0.01523 (19)	
C8	0.3873 (2)	0.85056 (5)	0.16229 (9)	0.0164 (2)	
C9	0.1586 (2)	0.89213 (5)	0.11465 (9)	0.0187 (2)	
H9A	0.0587	0.8876	0.0398	0.022*	
C10	0.0862 (2)	0.94063 (6)	0.18357 (10)	0.0197 (2)	
H10A	−0.0692	0.9702	0.1537	0.024*	0.254 (4)
C11	0.2396 (2)	0.94650 (6)	0.29702 (10)	0.0202 (2)	
H11A	0.1853	0.9801	0.3432	0.024*	0.746 (4)
C12	0.4672 (2)	0.90501 (6)	0.34381 (9)	0.0199 (2)	
H12A	0.5665	0.9094	0.4188	0.024*	
C13	0.5427 (2)	0.85640 (5)	0.27486 (9)	0.0163 (2)	
C14	1.5751 (2)	0.63299 (6)	0.36352 (9)	0.0208 (2)	
H14A	1.6860	0.5988	0.3405	0.031*	
H14B	1.4973	0.6156	0.4216	0.031*	
H14C	1.6918	0.6711	0.3916	0.031*	
C15	−0.1576 (3)	0.98865 (7)	0.13834 (13)	0.0201 (3)	0.746 (4)
H15A	−0.2119	0.9866	0.0577	0.030*	0.746 (4)
H15B	−0.1006	1.0336	0.1620	0.030*	0.746 (4)
H15C	−0.3142	0.9760	0.1668	0.030*	0.746 (4)
C15A	0.1355 (10)	0.9949 (2)	0.3651 (5)	0.0267 (11)	0.254 (4)
H15D	0.2593	0.9959	0.4395	0.040*	0.254 (4)
H15E	−0.0496	0.9820	0.3689	0.040*	0.254 (4)
H15F	0.1282	1.0387	0.3317	0.040*	0.254 (4)
H1N2	0.445 (4)	0.7809 (10)	0.0451 (16)	0.041 (5)*	
H1O1	1.058 (5)	0.7671 (11)	0.3717 (19)	0.058 (6)*	

### Atomic displacement parameters (Å^2^)

**Table:**

	*U*^11^	*U*^22^	*U*^33^	*U*^12^	*U*^13^	*U*^23^
O1	0.0181 (4)	0.0218 (4)	0.0123 (3)	0.0025 (3)	0.0008 (3)	−0.0007 (3)
N1	0.0156 (4)	0.0180 (4)	0.0141 (4)	0.0009 (3)	0.0025 (3)	0.0006 (3)
N2	0.0152 (4)	0.0189 (4)	0.0138 (4)	0.0011 (3)	0.0005 (3)	0.0000 (3)
C1	0.0143 (4)	0.0174 (4)	0.0126 (4)	−0.0020 (3)	0.0021 (3)	0.0010 (3)
C2	0.0149 (4)	0.0180 (4)	0.0164 (5)	−0.0004 (3)	0.0026 (3)	0.0014 (4)
C3	0.0183 (5)	0.0219 (5)	0.0196 (5)	0.0025 (4)	0.0037 (4)	−0.0019 (4)
C4	0.0211 (5)	0.0272 (5)	0.0181 (5)	0.0024 (4)	0.0030 (4)	−0.0046 (4)
C5	0.0174 (5)	0.0238 (5)	0.0137 (4)	0.0013 (4)	0.0007 (4)	−0.0014 (4)
C6	0.0142 (4)	0.0174 (4)	0.0133 (4)	−0.0003 (3)	0.0024 (3)	0.0001 (3)
C7	0.0136 (4)	0.0175 (4)	0.0136 (4)	−0.0005 (3)	0.0019 (3)	0.0018 (3)
C8	0.0148 (4)	0.0172 (4)	0.0163 (5)	−0.0010 (3)	0.0024 (3)	0.0008 (3)
C9	0.0157 (4)	0.0201 (5)	0.0184 (5)	0.0001 (4)	0.0010 (4)	0.0022 (4)
C10	0.0162 (4)	0.0194 (5)	0.0228 (5)	−0.0002 (4)	0.0037 (4)	0.0032 (4)
C11	0.0197 (5)	0.0195 (5)	0.0216 (5)	0.0008 (4)	0.0060 (4)	−0.0005 (4)
C12	0.0201 (5)	0.0220 (5)	0.0172 (5)	0.0007 (4)	0.0040 (4)	−0.0007 (4)
C13	0.0152 (4)	0.0174 (4)	0.0157 (5)	−0.0002 (3)	0.0027 (3)	0.0020 (3)
C14	0.0177 (5)	0.0234 (5)	0.0192 (5)	0.0033 (4)	0.0012 (4)	0.0023 (4)
C15	0.0167 (6)	0.0179 (7)	0.0243 (7)	0.0024 (5)	0.0027 (5)	0.0019 (5)
C15A	0.023 (2)	0.024 (2)	0.035 (3)	0.0009 (17)	0.0110 (19)	−0.0034 (18)

### Geometric parameters (Å, °)

**Table:**

O1—C1	1.3639 (12)	C9—H9A	0.9300
O1—H1O1	0.93 (2)	C10—C11	1.4100 (16)
N1—C7	1.3291 (13)	C10—C15	1.5217 (18)
N1—C13	1.3919 (13)	C10—H10A	0.9599
N2—C7	1.3706 (13)	C11—C12	1.3902 (15)
N2—C8	1.3842 (14)	C11—C15A	1.454 (5)
N2—H1N2	0.936 (19)	C11—H11A	0.9600
C1—C2	1.4045 (14)	C12—C13	1.3996 (15)
C1—C6	1.4124 (14)	C12—H12A	0.9300
C2—C3	1.3904 (15)	C14—H14A	0.9600
C2—C14	1.4990 (15)	C14—H14B	0.9600
C3—C4	1.3951 (16)	C14—H14C	0.9600
C3—H3A	0.9300	C15—H10A	0.5624
C4—C5	1.3877 (15)	C15—H15A	0.9600
C4—H4A	0.9300	C15—H15B	0.9600
C5—C6	1.4009 (14)	C15—H15C	0.9600
C5—H5A	0.9300	C15A—H11A	0.5030
C6—C7	1.4572 (14)	C15A—H15D	0.9600
C8—C9	1.3970 (14)	C15A—H15E	0.9600
C8—C13	1.4035 (14)	C15A—H15F	0.9600
C9—C10	1.3915 (16)		
			
C1—O1—H1O1	105.0 (14)	C9—C10—C15	120.98 (11)
C7—N1—C13	105.67 (8)	C11—C10—C15	118.62 (11)
C7—N2—C8	106.97 (9)	C9—C10—H10A	119.8
C7—N2—H1N2	127.1 (12)	C11—C10—H10A	119.8
C8—N2—H1N2	125.5 (12)	C12—C11—C10	121.86 (10)
O1—C1—C2	117.66 (9)	C12—C11—C15A	121.4 (2)
O1—C1—C6	121.79 (9)	C10—C11—C15A	116.6 (2)
C2—C1—C6	120.55 (9)	C12—C11—H11A	119.1
C3—C2—C1	118.62 (10)	C10—C11—H11A	119.0
C3—C2—C14	121.45 (10)	C11—C12—C13	118.11 (10)
C1—C2—C14	119.92 (9)	C11—C12—H12A	120.9
C2—C3—C4	121.60 (10)	C13—C12—H12A	120.9
C2—C3—H3A	119.2	N1—C13—C12	131.12 (10)
C4—C3—H3A	119.2	N1—C13—C8	109.24 (9)
C5—C4—C3	119.50 (10)	C12—C13—C8	119.63 (10)
C5—C4—H4A	120.2	C2—C14—H14A	109.5
C3—C4—H4A	120.2	C2—C14—H14B	109.5
C4—C5—C6	120.63 (10)	H14A—C14—H14B	109.5
C4—C5—H5A	119.7	C2—C14—H14C	109.5
C6—C5—H5A	119.7	H14A—C14—H14C	109.5
C5—C6—C1	119.07 (9)	H14B—C14—H14C	109.5
C5—C6—C7	121.12 (9)	C10—C15—H15A	109.5
C1—C6—C7	119.80 (9)	H10A—C15—H15A	107.6
N1—C7—N2	112.23 (9)	C10—C15—H15B	109.5
N1—C7—C6	123.58 (9)	H10A—C15—H15B	111.4
N2—C7—C6	124.17 (9)	C10—C15—H15C	109.5
N2—C8—C9	131.51 (10)	H10A—C15—H15C	109.4
N2—C8—C13	105.89 (9)	C11—C15A—H15D	109.5
C9—C8—C13	122.58 (10)	C11—C15A—H15E	109.5
C10—C9—C8	117.42 (10)	H15D—C15A—H15E	109.5
C10—C9—H9A	121.3	C11—C15A—H15F	109.5
C8—C9—H9A	121.3	H15D—C15A—H15F	109.5
C9—C10—C11	120.40 (10)	H15E—C15A—H15F	109.5
			
O1—C1—C2—C3	−178.69 (9)	C1—C6—C7—N2	−179.91 (9)
C6—C1—C2—C3	1.56 (15)	C7—N2—C8—C9	178.20 (11)
O1—C1—C2—C14	0.27 (14)	C7—N2—C8—C13	−0.17 (11)
C6—C1—C2—C14	−179.49 (9)	N2—C8—C9—C10	−178.36 (11)
C1—C2—C3—C4	−0.34 (17)	C13—C8—C9—C10	−0.22 (16)
C14—C2—C3—C4	−179.28 (10)	C8—C9—C10—C11	−0.31 (16)
C2—C3—C4—C5	−0.80 (18)	C8—C9—C10—C15	179.22 (11)
C3—C4—C5—C6	0.73 (17)	C9—C10—C11—C12	0.42 (17)
C4—C5—C6—C1	0.46 (16)	C15—C10—C11—C12	−179.13 (11)
C4—C5—C6—C7	179.02 (10)	C9—C10—C11—C15A	−175.2 (2)
O1—C1—C6—C5	178.63 (9)	C15—C10—C11—C15A	5.2 (3)
C2—C1—C6—C5	−1.62 (15)	C10—C11—C12—C13	0.03 (17)
O1—C1—C6—C7	0.06 (15)	C15A—C11—C12—C13	175.5 (2)
C2—C1—C6—C7	179.81 (9)	C7—N1—C13—C12	−178.89 (11)
C13—N1—C7—N2	−0.43 (12)	C7—N1—C13—C8	0.31 (11)
C13—N1—C7—C6	177.88 (9)	C11—C12—C13—N1	178.57 (10)
C8—N2—C7—N1	0.39 (12)	C11—C12—C13—C8	−0.56 (16)
C8—N2—C7—C6	−177.91 (9)	N2—C8—C13—N1	−0.08 (11)
C5—C6—C7—N1	−176.56 (10)	C9—C8—C13—N1	−178.63 (9)
C1—C6—C7—N1	1.98 (15)	N2—C8—C13—C12	179.22 (9)
C5—C6—C7—N2	1.55 (16)	C9—C8—C13—C12	0.67 (16)

### Hydrogen-bond geometry (Å, °)

**Table:**

*D*—H···*A*	*D*—H	H···*A*	*D*···*A*	*D*—H···*A*
N2—H1N2···O1^i^	0.936 (19)	2.095 (19)	2.9916 (12)	160.1 (18)
O1—H1O1···N1	0.93 (2)	1.74 (2)	2.6040 (12)	153 (2)
C15—H15C···Cg3^ii^	0.96	2.66	3.5731 (16)	160
C15A—H15F···Cg2^iii^	0.96	2.96	3.780 (4)	144

Symmetry codes: (i) *x*−1, −*y*+3/2, *z*−1/2; (ii) *x*−1, *y*, *z*; (iii) −*x*+1, *y*+1/2, −*z*+1/2.
